# Transcriptome analysis of roots from resistant and susceptible rice varieties infected with *Hirschmanniella mucronata*

**DOI:** 10.1002/2211-5463.12737

**Published:** 2019-10-22

**Authors:** Xiaotang Sun, Lei Zhang, Ziqing Tang, Xugen Shi, Jian Ma, Ruqiang Cui

**Affiliations:** ^1^ College of Agronomy/Key Laboratory of Crop Physiology, Ecology and Genetic Breeding Ministry of Education Jiangxi Agricultural University Nanchang Jiangxi China

**Keywords:** *Hirschmanniella mucronata*, mRNA‐seq, *Oryza sativa*, resistant variety, rice, transcriptome

## Abstract

*Hirschmanniella mucronata* is a plant‐parasitic nematode that is widespread in rice production areas and causes 10–25% yield losses a year on average. Here, we investigated the mechanism of resistance to this nematode by comparing the transcriptomes of roots from resistant (Jiabali) and susceptible (Bawangbian) varieties of rice. Of 39 233 unigenes, 2243. exhibited altered total expression levels between control and infected resistant and susceptible varieties. Significant differences were observed in the expression levels of genes related to stress, peptidase regulation or inhibition, oxidoreductase activity, peroxidase activity and antioxidant activity. The up‐regulated genes related to plant secondary metabolites, such as phenylpropanoid, lignin, cellulose or hemicellulose, may result in an increase in the degree of resistance of Jiabali to the *H. mucronata* infection compared with that of Bawangbian by affecting cell wall organization or biogenesis. Of the genes that responded similarly to *H. mucronata* infection, ~252 (~76.59%) showed greater changes (whether induced or suppressed) in RN155 (susceptible varieties infected by rice root nematode) than in RN51 (resistance varieties infected by rice root nematode). Nineteen pathogenesis‐related genes belonging to nine pathogenesis‐related gene families were significantly induced by *H. mucronata* in the infected roots of Jiabali and Bawangbian, and 13 differentially expressed genes showed changes in their abundance only in the susceptible Bawangbian variety. This study may help enhance our understanding of the mechanisms underlying plant resistance to nematodes.

**Abbreviations**
DAI: days after infection
DEG: differentially expressed gene
DR: defense response
ET: ethylene
FC: fold change
GO: gene ontology
JA: jasmonic acid
PR: pathogenesis‐related
Rboh: respiratory burst oxidase homolog
RGA: resistance gene analog
RNA‐seq: RNA sequencing
RPKM: reads per kilobase per million
RRN: rice root nematode
SA: salicylic acid
TO: trait ontology

Rice (*Oryza sativa*) is one of the most important food crops in the world and has proved to be a significantly excellent monocot model plant, but it is primarily grown in tropical regions with a hot and humid climate. Although the rice yield is affected by many types of pathogens, the plant‐parasitic nematode *Hirschmanniella mucronata*, an important rice pathogen, results in annual rice yield losses that range from 10% to 25% worldwide 1. One of the most damaging plant‐parasitic nematodes in the major rice‐growing regions is *Hirschmanniella oryzae*, which causes 58% of the damage to rice fields in the world and results in 25% yield losses 2.

The *Hirschmanniella* genus belongs to the Pratylenchidae and contains ~35 species. Some of them are among the largest Tylenchidae nematodes, and 11 species, including *H. oryzae*, *H. mucronata, H. spinicaudata, H. imamuri*, *H. mucronata*, *H. gracili*s, *H. thornei*, *H. caudacrena*, *H. mangaloriensis* *, H. shamimi* and *H. indica*, have been found to associate with *O. sativa* L. 3. The majority of these nematodes are migratory endoparasitic nematodes of plant roots 4. *H. mucronata* [the rice root nematode (RRN)] is the major rice‐parasitic nematode and the most common plant‐parasitic nematode found on irrigated rice 1. These organisms cause the necrosis of penetrated epidermal cells, and damage and destroy cortical cells, resulting in cavities in the cortex and necrotic regions, and roots that have been invaded turn yellowish brown and rot. Aboveground, the symptoms caused by RRN infection include growth retardation, decreased plant height, delayed tillering and weight reduction of dry matter. The juveniles and adults infect plant root tissues with the exception of the root tips and can move in the air channels between the radial lamellae of the parenchyma. A few days after entry, the female lays eggs that hatch in 4–5 days inside the roots. Under suitable conditions, the life cycle is completed in ~1 month. With the rice yield substantially increasing because of the recent changes in the practices of rice cultivation, the level of RRNs has been gradually increasing 5. Thus, research on treating RRN disease has become imperative. Currently, the control measures for the RRNs depend on varietal breeding for resistance, the application of nematicidal chemicals, crop rotation, fallow, tillage and mechanical disturbance, ammonia injection, thermal control and the use of a slow‐release rubber fertilizer formulation and organic manure to supply nutrition to the rice. The whole transcriptome sequencing [RNA sequencing (RNA‐seq)] approach has rarely been used to compare defense responses (DRs) in resistant and susceptible rice migratory nematode interactions.

Resistant and susceptible plants primarily have been used to detect the early stages of host–microorganism pathogen interactions. Gigante Vercelli and Vialone Nano, a durable rice blast‐resistant rice cultivar and a susceptible one, respectively, were used to dissect defense‐related genes, as well as the members of gene families that are involved in signal transduction and the regulation of pathogen‐related gene expression. The use of these two cultivars enabled the identification of genes encoding chitin oligosaccharide‐sensing factors, cell wall–associated kinases, mitogen‐activated protein kinase (MAPK) cascades and WRKY transcription factors that were critical to Gigante Vercelli resistance during the early steps of defense perception signaling 6. A genome‐wide analysis of the gene families of resistance gene analogs (RGAs) was conducted in *Gossypium raimondii*, a *Verticillium* wilt‐resistant cotton species, and the analysis identified plant RGAs that served as resistance gene candidates and were involved in cotton DR. These genes formed 26 *Verticillium dahliae* response loci inside RGA gene‐rich clusters after inoculation with *V. dahliae* 7. In the current study on rice, genome‐wide, expression, phylogenetic and synteny analyses were conducted for resistance genes and DR genes primarily by the use of three species of rice: *O. sativa* ssp. *indica*, *O. sativa* ssp. *japonica* and *O. brachyantha* 8. The jasmonic acid (JA) and salicylic acid (SA) biosynthetic pathways were demonstrated to be a prerequisite for defense against *Meloidogyne graminicola*, and abscisic acid was found to play a negative role in rice defense 9. In this study, this was the first use of RRN to explore resistance and DR genes using mRNA‐seq in the resistant rice cultivar Jiabali and the susceptible rice cultivar Bawangbian.

## Materials and methods

### Preparation of nematodes

*H. mucronata* was collected from irrigated rice fields in Jiangxi Province, China. The nematodes were extracted and cultured as described by Kyndt *et al.* 10. Ten rice varieties were obtained from the Key Laboratory of Crop Physiology, Ecology and Genetic Breeding, Ministry of Education, including ‘Baoxuan21’, ‘Guichao2’, ‘Taizhong65’, ‘Wuyunjing3’, ‘Wuyunjing7’, ‘Zhenxian232’ and ‘Zhenxian97B’, which are improved cultivars, and ‘Bawangbian’, ‘Mamagu’ and ‘Jiabali’, which are local varieties. The experiments were conducted in greenhouse conditions with an average air temperature ranging from 28 °C to 30 °C and relative humidity ranging from 85% to 90%.

### Screening resistant varieties

Three rice seedlings of each variety were inoculated in plastic pots (10 cm in diameter and 15 cm in height) at the seedling stages with 400 nematodes per plant 2. The trials were repeated three times with three biological replicates. After 30 days, the roots were removed, washed and examined for RRN.

### Plant material and RNA preparation

Bawangbian and Jiabali rice seeds were germinated for 4 days at 30 °C, transferred to synthetic absorbent polymer substrate 11 and grown further in a manual climatic box. Fifteen‐day‐old rice plants were inoculated with 400 *H. oryzae* nematodes per plant. All of the control plants were mock inoculated. Control and infected tissues were collected at 7 days after infection (DAI) as described by Kyndt *et al.* 12. Three biological replicates were used with each treatment, and each replicate contained a pool of three different plants. Notably, for mRNA‐seq, root tissue of the resistant and susceptible varieties was collected and marked R51 and R155, respectively, and collected root tissue infected by RRN from the resistant and susceptible varieties was marked RN51 and RN155, respectively. Total RNA was extracted using an RNeasy Plant Mini Kit (Qiagen, Venlo, the Netherlands). The quantity and quality of RNA were checked and measured using a NanoDrop 2000 spectrophotometer (Thermo Fisher Scientific Inc., Wilmington, DE, USA). RNA samples were sent to Novogene Bioinformatics Technology Co.(Tianjin, China) for RNA‐seq library preparation using a TruSeq SBS Kit (Illumina Inc., San Diego, CA, USA) and paired‐end sequencing using an Illumina Hi‐Seq 2000 platform (Illumina).

### Mapping reads to the reference genome and annotated transcripts

The read quality was further assessed using FastQC application, and reads containing contaminant primer/adapters and long stretches of poor‐quality bases were removed, thus producing clean reads. After downloading the reference genome (*O. sativa* L. spp. *japonica* cv. *Nipponbare*) and gene model annotation, the reads were mapped with bowtie v2.0.6, tophat v2.0.9 13 and cufflinks v2.1.1 14.

### Identification of novel transcripts

The cufflinks Reference Annotation Based Transcript assembly method was used to construct and identify both known and novel transcripts from the tophat alignment results, and the novel transcripts were labeled.

### Quantification and analysis of differential gene expression

The read counts mapped to each gene were collected using htseq v0.6.1, and to define gene expression, we calculated the reads per kilobase per million (RPKM) of each gene based on the length of the gene and read counts mapped to this gene. RPKM simultaneously eliminates the effect of sequencing depth and gene length for the read counts. In addition, genes greater than the 0.1 RPKM threshold were considered to be expressed. Differential expression analysis of the genes in two conditions (two biological replicates per condition) was performed using the deseq r package v1.10.1 15 because an r package implementing a model based on negative binomial distribution was developed to specifically adapt to biological variance. The resulting *P* values were adjusted with the Benjamini and Hochberg approach to control the false discovery rate. Genes with an adjusted *P* < 0.05 found by deseq were assigned as being differentially expressed. For all further analyses, the expression level of each transcript per condition was estimated as the average number of reads detected across the biological replicates. The fold change (FC) of each transcript is calculated as the ratio of the average number of reads +1 (to avoid 0 values) in the different conditions, and the FC values were then transformed into log_2_.

### Enrichment analysis of differentially expressed genes

Gene ontology (GO) analysis and GO enrichment were implemented using the goseq r package 16, in which any gene length bias was corrected. A PAGE 17 was executed based on the log_2_FC of the transcripts. The Benjamini and Hochberg false discovery rate correction was performed using the default parameters to adjust the *P* value. GO terms with corrected *P* < 0.05 were considered to delineate significant enrichment by differentially expressed genes (DEGs). Kyoto Encyclopedia of Genes and Genomes (KEGG) mapping was used to determine the primary metabolic and signal transduction pathways of the DEGs, and kobas software was used to test the statistical enrichment of DEGs in the KEGG pathways. Moreover, to recognize the functions of unknown genes or the unknown functions of genes identified, we collected genes embracing different or similar expression patterns into a cluster through hierarchical clustering based on the RPKM of the DEGs of each treatment.

Mercator (http://www.plabipd.de/portal/mercator-sequence-annotation) was employed to classify the function of the DEGs 18. In addition, the gene names and trait ontology (TO) were added to the Oryzabase database (https://shigen.nig.ac.jp/rice/oryzabase/download/gene). Also, clusterprofiler 19 was used to enrich the TO of the DEGs. The network was obtained using cytoscape v3.7.1 20.

### Quantitative real‐time PCR analysis

For quantitative real‐time PCR analysis, first‐strand cDNAs were synthesized from DNase I‐treated total RNA using the SuperScript® IV First‐Strand Synthesis System according to the manufacturer’s instructions. Quantitative real‐time PCR was performed in an optical 96‐well plate with an ABI 7500 Real‐Time PCR System. Each reaction contained 10 μL of 2× SYBR Green Master Mix reagent (Applied Biosystems, Thermo Fisher Scientific Inc., Wilmington, DE, USA), 2.0 μL of cDNA samples and 200 nm each of the gene‐specific primers in a final volume of 20 μL. The thermal cycle used was as follows: 95 °C for 2 min; 40 cycles of 95 °C for 15 s, 60 °C for 34 s, and 72 °C for 30 s. All gene‐specific primers for quantitative real‐time PCR are listed in Table S1 and designed on the basis of the cDNA sequences. Actin was selected as the housekeeping gene. The specificity of the reactions was checked by melting curve analysis, and three replicates of each cDNA sample were used for quantitative real‐time PCR analysis.

## Results and Discussion

### Isolation of resistant varieties

To obtain the rice variety with resistance to RRN, we inoculated 15‐day‐old rice plants with ~400 RRNs per plant in the greenhouse. Among the ten rice varieties studied (Table 1), the number of nematodes was the fewest in the Jiabali roots, whereas most were found in the Bawangbian roots. Thus, Jiabali and Bawangbian were selected as the resistant and susceptible varieties, respectively, in the following experiments.

**Table 1:** Nematode number of different varieties per gram root.

Name of variety	RRNs per gram root
Taizhong65	16.2 ± 2.8^a^
Wuyunjing3	3.6 ± 0.9^b^
Zhenxian97B	1.2 ± 0.5^b^
Jiabali	0.1 ± 0.1^b^
Mamagu	18.2 ± 1.6^a,c^
Baoxun21	19.2 ± 5.0^a,c^
Guichao2	21.4 ± 6.6^a,c^
Wuyunjing7	24.1 ± 6.8^c^
Bawangbian	44.8 ± 17.0^d^
Zhenxian232	6.4 ± 2.2^b^

^a,b,c,d^ Data are means (*n* = 9) of three independent experiments ± SD; means with different letters (a, b, c or d) are significantly different at *P* < 0.05 (Duncan’s multiple range tests).

### Overview of the RNA‐seq data

To investigate the possible molecular response mechanism to *H. mucronata* in the roots of the resistant (Jiabali) and susceptible (Bawangbian) varieties, we conducted a transcriptome analysis of their roots at 7 DAI. A total of 141 119 236 and 123 087 638 reads were acquired from the infected (RN155 and RN51) and uninfected (R155 and R51) roots of Bawangbian and Jiabali varieties, respectively, and 75.49% and 79.58% of them were mapped to the whole reference genome sequence of *O. sativa* L. spp. *japonica* cv. *Nipponbare* (Table S2). The total length of the mapped reads was ~10.65 and 9.80 billion bases, representing 26.88‐fold and 24.72‐fold of the rice genome size, respectively (Table S2). In addition, ~39 233 rice unigenes were detected in the roots.

tophat and cufflinks were used to detect potential exon junctions and unannotated transcribed regions. The locations of the resulting new transcriptional active region (nTARs) were matched against the *O. sativa* MSU6 genes. All new transcripts overlapping known genes were excluded, resulting in 968 putative nTARs remaining (Table S3).

**Figure 1:**
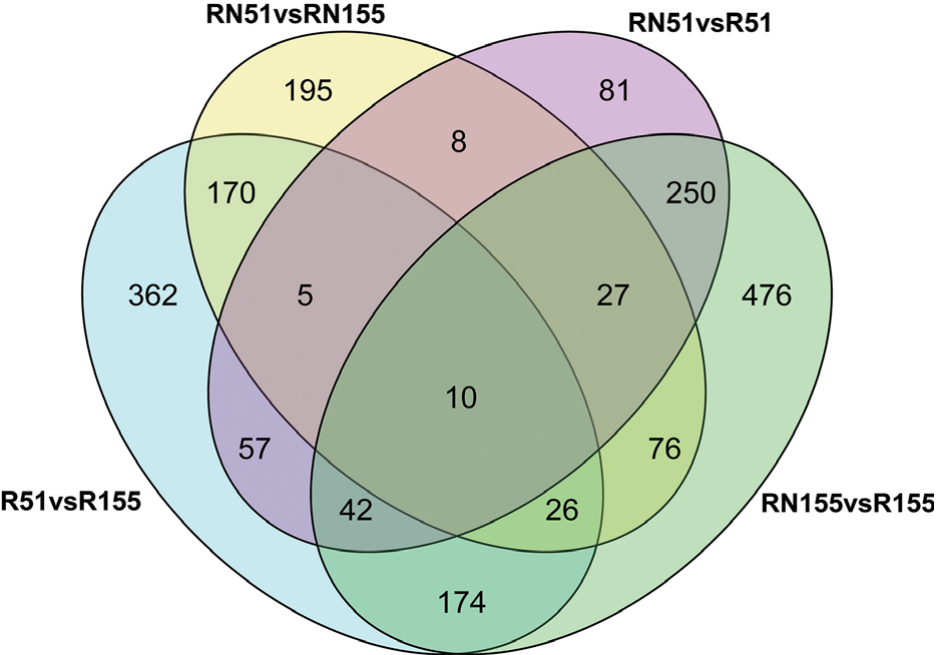
The number of different expression genes among the four comparative groups.

**Figure 2:**
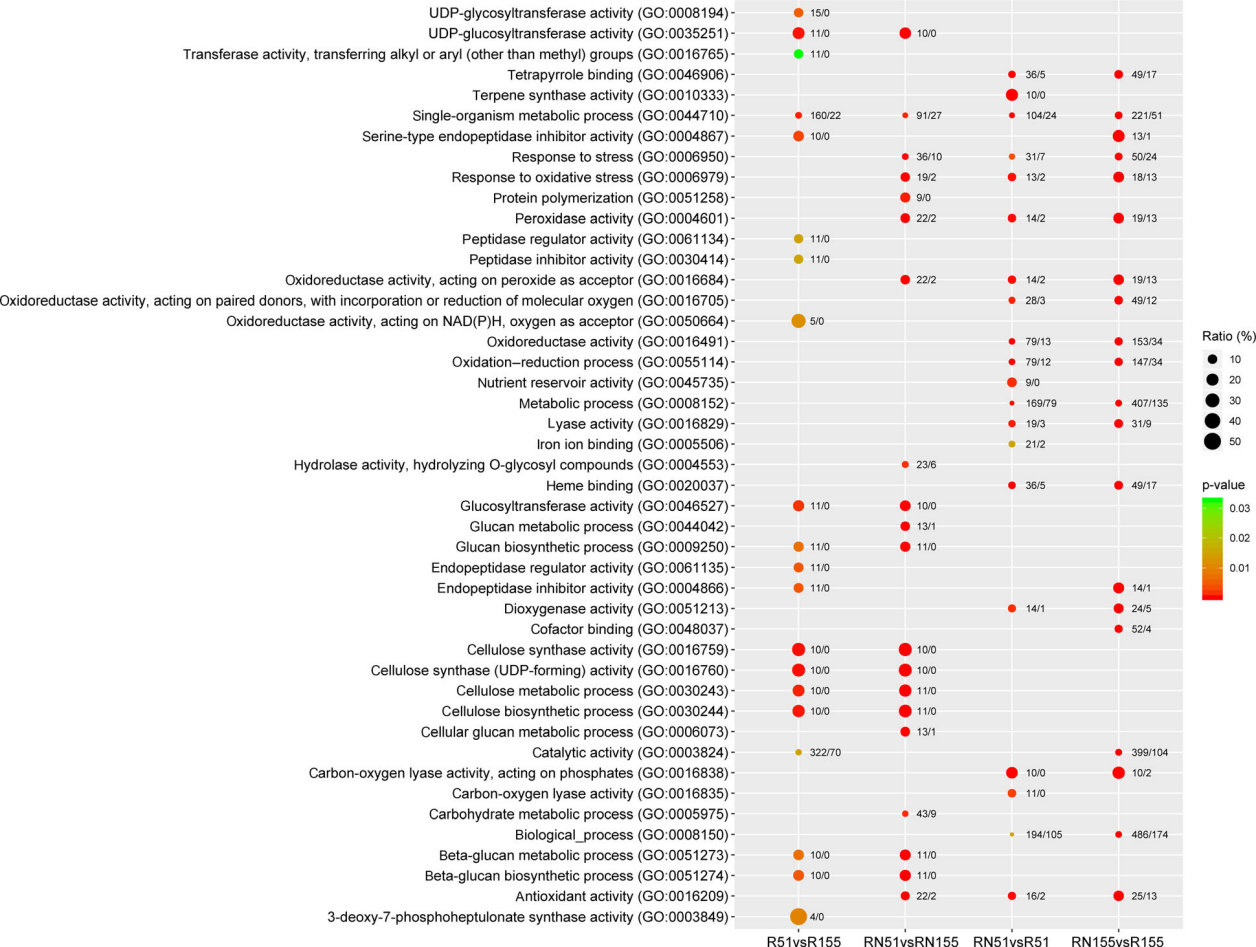
GO annotation of the DEGs from the roots of the resistant and susceptible varieties at 7 DAI. The top 20 GO annotations with *P* < 0.05 are shown here, and more information about the GO annotations is shown in Table S4. Points are the percentage of the number of DEGs (ratios of the number of DEGs to the total number of detected genes of the same GO terms) in the paired transcriptome analysis. The numbers of up‐regulated DEGs are on the left of backslashes, and down‐regulated DEGs are on the right. vs, versus.

To obtain candidate response genes to the nematode infection in the resistant and susceptible varieties, we screened DEGs (|log_2_FC| ≥ 1, *P* ≤ 0.05) from the roots at 7 DAI. There were ~2243. DEGs (1883 known and 76 novel genes) and ~846, 1081, 480 and 517 in R51 versus R155, RN155 versus R155, RN51 versus R51 and RN51 versus RN155, respectively (Fig. 1 and Table S4). According to the Gene Set Enrichment analysis of the DEGs, ~124 GO terms were significantly enriched, and 22, 63, 29 and 66 of the GO terms in R51 versus R155, RN155 versus R155, RN51 versus R51 and RN51 versus RN155, respectively, were enriched (Fig. 2 and Table S5).

### Native transcriptome differences in the roots of the resistant and susceptible hosts

**Figure 3:**
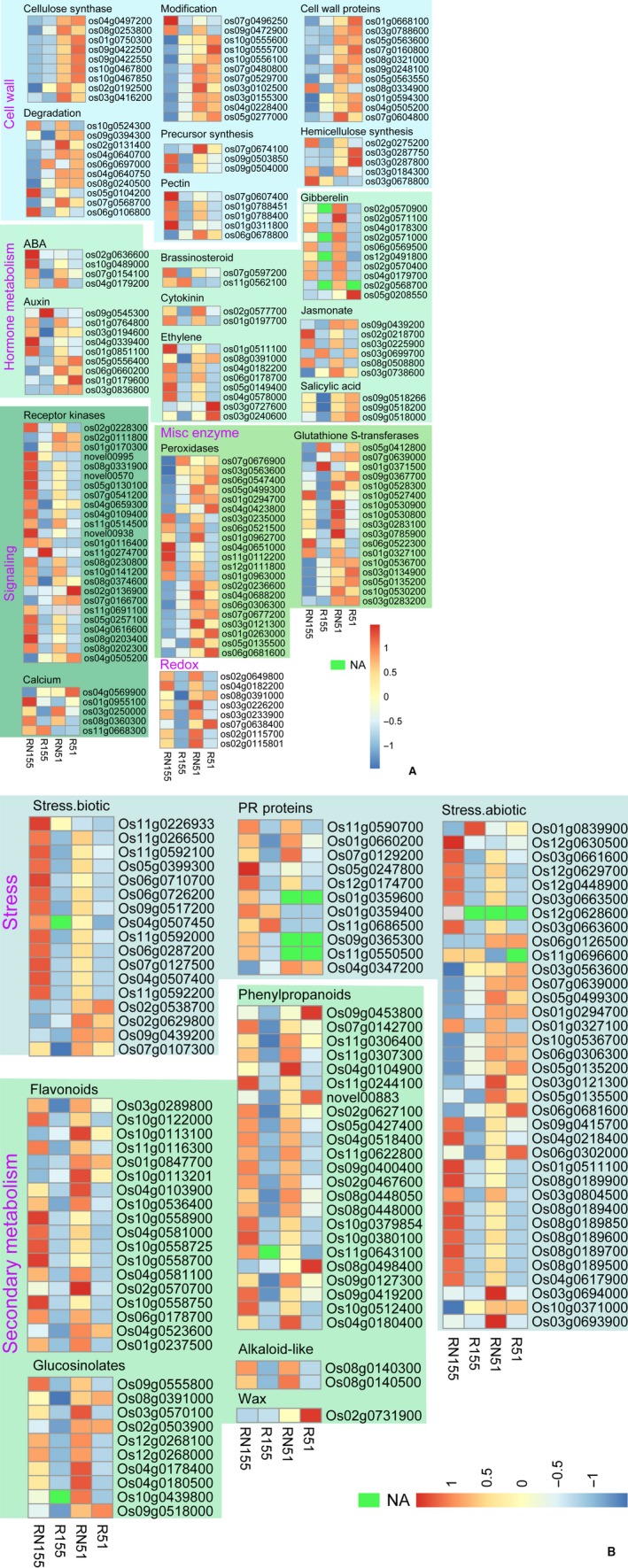
Expression profile of the DEGs in roots based on RNA‐seq. The DEGs were annotated according to MapMan (https://mapman.gabipd.org/). The DEGs with |log_2_FC| ≥ 2 are shown. Different pathways are in different colors. Fragments per kilogram per million are centered and scaled in the row direction, and the heatmap was drawn using pheatmap v1.0.10 (r package). The gene information is detailed in Table S5. Misc, miscellaneous; NA, not available and in green. (B) is the continued Figure of (A).

To investigate the different native genes of the Jiabali (resistant) and Bawangbian (susceptible) varieties in roots under the same conditions, we analyzed their root transcriptomes. According to the GO annotations of the DEGs, the genes involved in cellulose biosynthetic and metabolic process, beta‐glucan biosynthetic and metabolism, glucan biosynthetic and metabolic process, regulator process of peptidase activity and oxidation‐reduction process were significantly up‐regulated (Fig. 2 and Table S5). For example, compared with the susceptible variety, the genes related to cellulose or hemicellulose synthesis were more highly expressed in resistant varieties and included *OsBC1*, *OsCesA1*, *OsCesA4*, *OsCesA6*, *OsCesA7*, *OsCesA8*, *OsGT43B* and *OsGT43I* (Fig. 3 and Table S5). In addition, the gene expression levels of cell wall protein (*OsFLA3*, *OsFLA6*, *OsFLA7*, *OsFLA18* and *OsFLA24* and two members of the LRR gene family) were higher in the Jiabali roots than those of the Bawangbian (Fig. 3 and Table S4). In the cell wall degradation process, there were eight up‐regulated genes related to beta‐1,4‐glucanases; *OsGH9B11* was the strongest (log_2_FC = 2.04) (Fig. 3 and Table S4). Only *OsEXPB2* (log_2_FC = 1.37) was significantly induced during the cell wall modification process (Fig. 3 and Table S4). In the early (3 DAI) or late (7 DAI) process of *M. graminicola* inoculation in *O. sativa* L. spp. *japonica* cv. *Nipponbare* (GSOR‐100, US Department of Agriculture), which is a susceptible rice cultivar, the GO terms ‘plant‐type cell wall organization’ and ‘biogenesis’ were down‐regulated in the infected roots 12. Although our results indicated that some important genes in the process of cell wall organization or biogenesis were expressed at significantly higher levels in the resistant roots, this change suggests that a strong degree of variability in cellulose or hemicellulose synthesis may result in greater resistance to nematode infections.

In the hormone metabolic pathways, jasmonate, ethylene (ET) and SA can induce systemic defense against *M. graminicola* in rice (*O. sativa*), and the JA pathway is a key player in the systemically induced defense against the rice root‐knot nematode 21. In a comparison of the root transcriptome of Bawangbian with that of Jiabali, all eight genes detected were related to JA; one gene related to the JA signal transduction (*OsJAZ7*) and seven genes to JA biosynthesis, such as *OPR1*, *OPR5*, *AOS* and *AOC*, were significantly up‐regulated in the roots of the resistant variety (Fig. 3 and Table S4). In addition, JA biosynthesis is required for ET‐induced systemic defense, and ET can activate JA biosynthesis 21. Compared with the susceptible variety, three ET biosynthetic genes, *Os08g0391000* (log_2_FC = 3.61), *OsACS1* (log_2_FC = 2.02) and *OsCBSX2* (log_2_FC = 1.40), were more highly expressed, whereas two biosynthetic genes, *OsACO4* (log_2_FC = −1.16) and *Os11g0187001* (log_2_FC = −1.13), and an ET degradation gene, *Os01g0934500* (log_2_FC = −1.12), were down‐regulated (Fig. 3 and Table S4).

SA is believed to regulate defense against biotrophic pathogens, whereas JA and ET synergistically operate to manage necrotrophic pathogens and insect resistance 22. All three genes detected that were involved in the SA pathway, *OsUGT74H4* (log_2_FC = 4.12), *OsSGT* (log_2_FC = 2.56) and *Os09g0518266* (log_2_FC = 2.52), were significantly expressed more highly in R51 than in R155 (Fig. 3 and Table S4). Gibberellin plays important roles in the susceptibility of rice to *M. graminicola* infection 23. *M. graminicola* infection in rice induces strong transcriptional changes in the gibberellin (GA) biosynthesis (*GA20ox1* and *GA20ox2*) and signaling (*GID1L2*) genes in galls (3 and 7 DAI) and giant cells (7 and 14 DAI) 12, 24. *GID1L2* (log_2_FC = 2.66) in R51 was expressed more highly than that in R155, and there was no transcriptional change for *GA20ox1* in R51 and R155, whereas *Os05g0208550*, similar to gibberellin 2‐beta‐dioxygenase and relative to GA catabolism, was expressed at a higher level (log_2_FC = 2.34) (Fig. 3 and Table S4).

GA‐induced nematode susceptibility is largely independent of auxin biosynthesis but relies on auxin transport, and the auxin transport proteins are targeted by plant‐parasitic nematodes to channel the auxin necessary for the development of their feeding structure 23, 25, 26, 27. There were ten DEGs relating to auxin, and only the expression of *OsSAUR39* (log_2_FC = −2.74) (Fig. 3 and Table S4) that acts as a negative regulator of auxin synthesis and transport in rice 28 was significantly lower in R51 than in R155, whereas the other nine DEGs were more highly expressed in R51 (Fig. 3 and Table S4). For example, the expression of *OsGH3‐8* is ~2.54‐fold higher in R51 than in R155, and it is an auxin‐responsive gene that activates disease resistance in a SA signaling– and JA signaling–independent pathway 29. *Os03g0836800*, an indole‐3‐acetic acid amidohydrolase, was expressed more highly in R51 than in R155 (log_2_FC = 7.26) (Fig. 3 and Table S4), and this gene may play important roles in metabolic level cross talk between the indole‐3‐acetic acid and JA signaling pathways under stress conditions 30.

Aside from the relation of the DEGs described earlier to phytohormone metabolism in the comparison of the uninfected root transcriptome between Jiabali and Bawangbian varieties, there were only two down‐regulated DEGs involved in brassinosteroid metabolism, including *OsIAS* (log_2_FC = −2.67) related to brassinosteroid synthesis (Fig. 3 and Table S4). Nahar *et al.* 31 found that brassinosteroids suppress rice defense against root‐knot nematodes through antagonism with the JA pathway. In migratory nematode‐infected roots, the cytokinin pathway was not strongly influenced, and a strong suppression was detected in roots 3 DAI 12. According to these results, only *OsCKX11* was involved in the degradation of cytokinins with a 1.79‐fold reduction (Fig. 3 and Table S4).

Plant hormones have pivotal roles in the regulation of immune responses to microbial pathogens, insect herbivores and beneficial microbes. Their signaling pathways are interconnected in a complex network, which provides plants with an enormous regulatory potential to rapidly adapt to their biotic environment and to use their limited resources for growth and survival in a cost‐efficient manner 22. Based on our results, genes in the phytohormone immunity to nematodes were different in the transcriptomic level in the roots of the resistant and susceptible varieties, and that is another reason for the enhanced resistance of Jiabali to *H. mucronata*.

In the signal transduction pathway, ~43 receptor kinases genes, such as leucine‐rich repeat, calcium and G proteins, were significantly differentially expressed in R51 and R155. Among them, a strong up‐regulation was detected in the transcripts encoding *OsRMC* (Os04g0659300), *IREN* (Os10g0203000), *OsRLCK303* (Os10g0516200) and *OsCaM1* (Os03g0319300) (Fig. 3 and Table S4) in the uninfected roots of the Jiabali variety compared with those of the Bawangbian variety.

**Figure 4:**
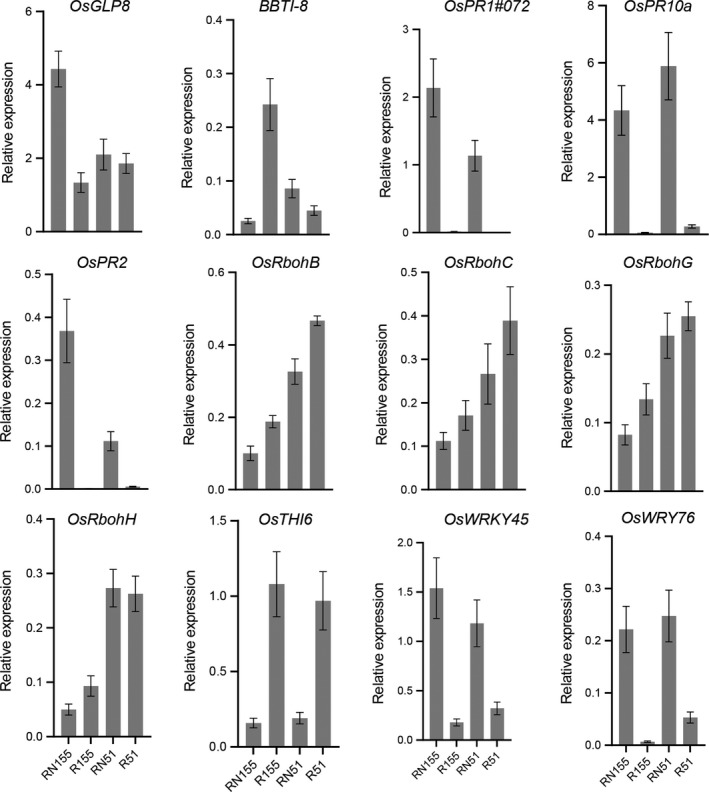
Expressions of some genes through quantitative real‐time PCR. The housekeeping gene was *ACTIN*. The bars were the average relative expressions, and error bars were the standard deviation (*n* = 3).

There were 26 DEGs related to biotic stress in the R51 versus R155 group, and 17 of the 26 genes were expressed more highly in the uninfected roots of R51 than in R155. For example, the chitin‐responsive NADPH oxidases *OsRbohB* (Os01g0360200), *OsRbohH* (Os12g0541300), *OsRbohC* (Os05g0528000) and *OsRbohG* (Os09g0438000), and the pathogenesis‐related (PR) proteins OsPR1‐101 (Os10g0191300) and OsPR4c/d (Os11g0592000/Os11g0591800) were more highly expressed in the uninfected roots of R51 than in R155 (Figs 3 and 4 and Table S4). Rice plasma membrane microdomains that play an important role in the resistance to rice blast fungus infection are required for the dynamics of the Rac/ROP small GTPase Rac1 and respiratory burst oxidase homologs (Rbohs) in response to a chitin elicitor, and Os‐Rbohs are essential for resistance to the rice blast fungus 32 and to *H. mucronata*.

Many genes involved in plant secondary metabolism, such as phenylpropanoid, lignin and flavonoids, were affected after nematode infection 10, 33, 34, 35. The phenylpropanoid or lignin pathway and its products play important roles in the incompatible interaction and cultivar resistance against nematodes 33. Compared with R155, all 19 DEGs involved in phenylpropanoid (7 DEGs) or lignin (12 DEGs) metabolism were significantly up‐regulated in the uninfected roots of Jiabali (Table S4). The expression of *OsPAL1* (Os02g0627100) was up‐regulated 1.43‐fold compared with that of R155. In the rice‐resistant cultivar Manikpukha, *OsPAL1* plays a pivotal role in resistance to the rice stem nematode *Ditylenchus angustus*, and the lignin content significantly increased after the nematode infection 33. That showed that rice resistance to the nematode partially relied on the metabolism of phenylpropanoid or the biosynthesis of lignin. The higher expression of genes related to the biosynthesis of lignin in the Jiabali variety may be one reason this variety is significantly more resistant to *H. mucronata* than is the Bawangbian variety.

### Resistance and susceptible host response to *H. mucronata* infection 1 week after nematode inoculation

There were ~480 and 1081 DEGs in the infected Jiabali and Bawangbian roots, respectively. The expression of 329 DEGs was significantly variable in both RN51 and RN155 compared with their mock treatments (Table S6). Approximately 263 of the 329 DEGs were up‐regulated, and 60 were down‐regulated in the roots of the two varieties at 7 DAI by *H. mucronata*. Those genes may play important roles in the response of hosts to nematodes but may not be relevant to the rice genotype. In addition to these genes, 112 DEGs were down‐regulated and 39 up‐regulated solely in the Jiabali‐infected roots. However, 258 DEGs were down‐regulated, and 494 were up‐regulated solely in the infected Bawangbian roots (Table S6). In addition, their response to *H. mucronata* may be related to the genotype. Six genes, including *OsIDI4* (Os09g0453800), *OsWD40‐193* (Os12g0132400), Os11g0134950, *OsTOM1* (Os11g0134900), *OsZIFL9* (Os12g0132500) and *OsTHI3* (Os06g0513050), show a different model of response in both rice genotypes, and the expression of the first five genes was suppressed, whereas the last one was induced in RN51, but not in RN155 (Table S6). Interestingly, among the genes with the same response to *H. mucronata*, there were ~252 (~76.59%) genes, and the change in their levels of expression induced or suppressed by *H. mucronata* was higher in RN155 than in RN51.

According to the GO annotation, the expression of stress‐related genes, including peptidase regulation or inhibitor, oxidoreductase activity, peroxidase activity and antioxidant activity, was significantly changeable, and most of their levels of expression in the enriched DEGs were up‐regulated (Fig. 2).

**Figure 5:**
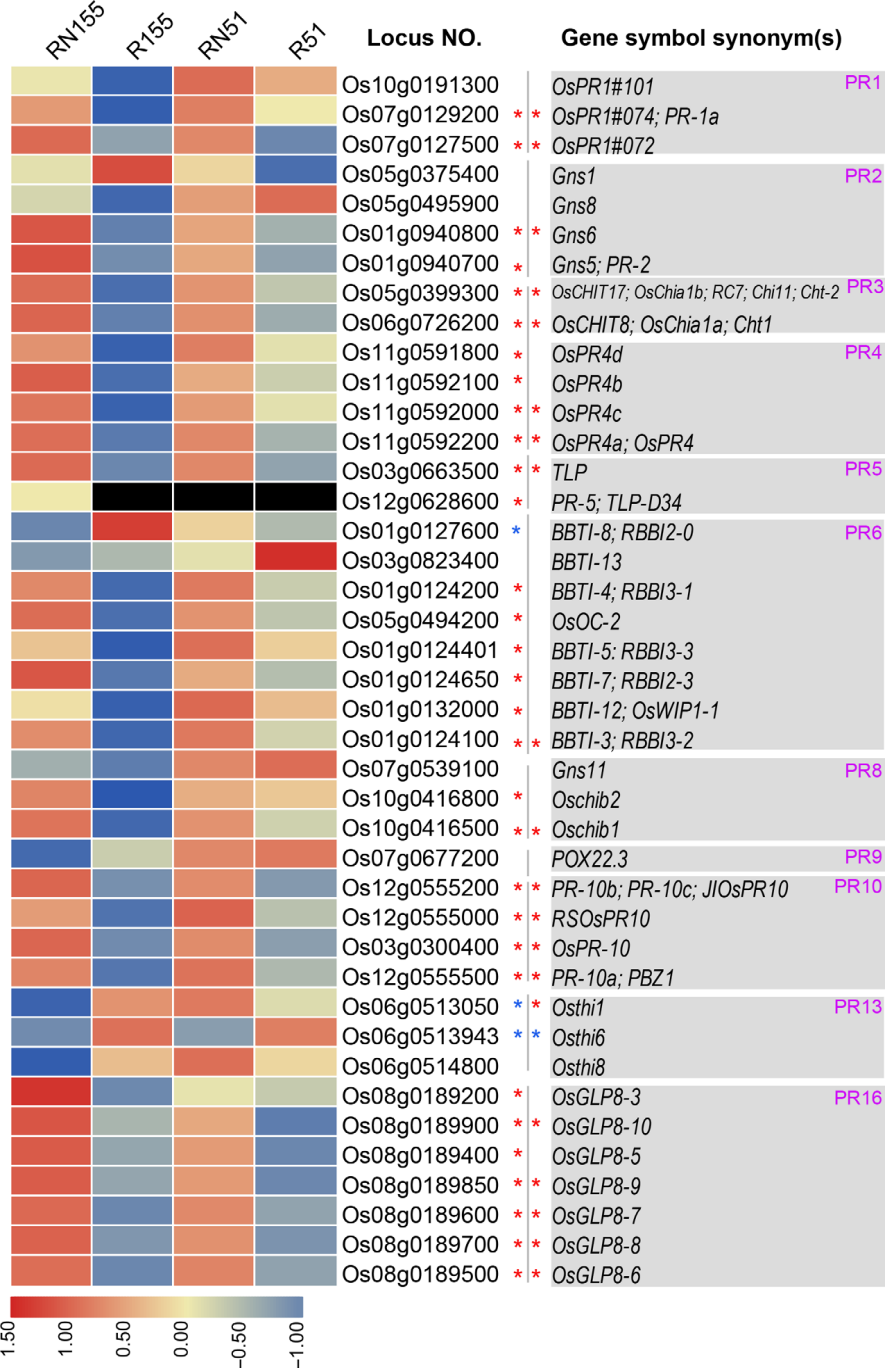
Responses of the PR genes to *H. mucronata.* Fragments per kilogram per million (FPKMs) of the genes are scaled in the row direction, and the heatmap was drawn using pheatmap v1.0.10 (r package). Asterisks indicate genes were significantly up‐ (in red) and down‐regulated (in blue) in RN155 (on the left of gray lines) or RN155 (on the right of gray lines). Letters in purple indicate the PR gene family. Filled color is in black, and the FPKMs of the genes were zero. Black，no significant difference.

In rice, there are ~12 known PR gene families with 113 members, and they have been reported to be consistently induced by bacterial, fungal and nematode infection at the mRNA or protein levels 36, 37, 38, 39, 40, 41, 42, 43. In this study, there were 41 DEGs belonging to 11 PR gene families (Figs 3 and 5). The expression of 34 DEGs was significantly changeable in the infected roots of both Jiabali and Bawangbian. Approximately 19 out of 41 DEGs were significantly induced by *H. mucronata* in the infected roots of Jiabali and Bawangbian varieties, and 13 DEGs showed changes in their abundance only in the susceptible Bawangbian. For example, the expression of *OsPR1#072* (Os07g0127500) had the greatest magnitude of change in the infected roots of Jiabali (log_2_FC = 9.06) and Bawangbian (log_2_FC = 7.26) (Fig. 4) varieties, and the other two PR genes, *OsPR1#101* and *OsPR1#074* (PR‐1a), were significantly up‐regulated in the infected roots. *PR‐1*, which is often used as a robust marker for SA‐responsive gene expression, has been frequently used as a marker gene for systemic acquired resistance in many plant species 22, 44. OsPR1a, OsPR1b and OsPR10a were significantly induced at the protein level by *Xanthomonas oryzae* pv. *oryzae* in the late stages of inoculation 38. All four members (Os03g0300400, Os12g0555200, Os12g0555000 and Os12g0555500) of the *PR10* gene family were all significantly induced in the infected roots of the two varieties (Figs 4 and 5). The *PR10* genes were not only induced by biotic stress but also by abiotic stress. For example, the overexpression of *JIOsPR10* (Os12g0555200) can reduce the susceptibility to the rice blast fungus and enhance salt and drought stress tolerance compared with the wild‐type, and *JIOsPR10* plays important roles in biotic and abiotic stress tolerance presumably by the activation of stress‐related proteins^45^. Transgenic rice and *Arabidopsis*‐overexpressing *OsPR10a* (Os12g0555500) (Fig. 4) significantly increased the length of the primary root under phosphate deficiency conditions. These results showed that *OsPR10a* might play multiple roles in phosphate recycling in phosphate‐starved cells and senescing leaves, and could improve resistance to pathogen infection and/or against chewing insect pests. It is possible that phosphate acquisition or homeostasis is associated with plant disease resistance 37.

Among the three DEGs, only *OsTHI6* (Os06g0513943), one member of the *PR13* gene family, was significantly suppressed by *H. mucronata* in the two varieties (Fig. 4). Thionins are antimicrobial peptides that are involved in plant defense 46. The expression of *OsTHI1*, *OsTHI2* and *OsTHI7/6* is significantly suppressed by *M.* *graminicola* in galls compared with the control root tips until 7 DAI when the giant cells mature*. *Even at 14 DAI, *OsTHI7/6* is still expressed at lower levels in the galls than in the control root tips under the conditions of *M. graminicola* infection, although this difference is not significant. In addition, the overexpression of *OsTHI7* can improve the resistance of rice to *M. graminicola* 36. This improvement in resistance implies that *OsTHI6* has an important role in improving the resistance of rice to *H. mucronata.* However, *OsTHI1* and *OsTHI2* are strongly induced by *M. graminicola* at 14 DAI 36. In our results, *OsTHI1* (Os06g0513050) was significantly induced in the Jiabali variety, but significantly suppressed in the Bawangbian variety (Fig. 5). This finding implies that *OsTHI1* responded differently to *H. mucronata* in different genotypes. *BBTI‐8* (Os01g0127600) was suppressed by *H. mucronata* only in Bawangbian and showed no detectable difference in expression in Jiabali. However, the expression of *PR5* was detected only in the infected roots of Bawangbian and not in the tissues of RN51, R51 and R155 (Fig. 5).

Throughout evolution, plants have developed a sophisticated immune system to protect themselves from invading pathogens. Plant hormones that serve as cellular signal molecules have pivotal roles in the regulation of immune responses to fungal, bacterial and nematode infection 22, 47, 48. Their signaling pathways are interconnected in a complex network, which provides plants with an enormous regulatory potential to rapidly adapt to their biotic environment and to use their limited resources for growth and survival in a cost‐efficient manner 22.

In this study, 58 DEGs relative to hormone metabolism responded to the infection of *H. mucronata* in the roots of Jiabali and/or Bawangbian (Fig. 2 and Table S4). Infection with *H. mucronata* resulted in the significant up‐regulation of the expression of 15 out of the 58 DEGs in the infected roots of both Jiabali and Bawangbian, and most of them were related to hormone biosynthesis, such as gibberellin (7), JA (2), ET (3), cytokinin (1) and abscisic acid (1). Only *OsEBP‐89* (Os03g0182800) was related to the signal transduction of ET. For the resistant Jiabali variety, only five genes (two genes related to auxin‐responsive protein, one to ET synthesis and two to ET signal transduction) were particularly suppressed, and two genes relative to gibberellin synthesis were induced by *H. mucronata* in the roots. These genes had important roles in the resistance response of the hosts. For example, only 8 genes were particularly suppressed and 28 genes were induced by *H. mucronata* in the roots of the susceptible variety Bawangbian (Fig. 2 and Table S4), and these genes could be related to the susceptible response of the hosts. These results revealed that hormone metabolism had a greater degree of change in the susceptible than the resistant host. Among the genes related to plant hormones, the expression of the two genes *OsCPS4* (Os04g0178300) and *OsKS4* (Os04g0179700) related to gibberellin synthesis was mostly induced by *H. mucronata* in the infected roots of Bawangbian (log_2_FC = 10.61) and Jiabali (log_2_FC = 9.73), respectively. In contrast with the observations from *M. graminicola* and rice interactions, gibberellin plays a dominant role over JA in determining the susceptibility to *M. graminicola* in rice 23. It could be that gibberellin has important roles in the interaction between rice and *H. mucronata*.

### TO network of DEGs

**Figure 6:**
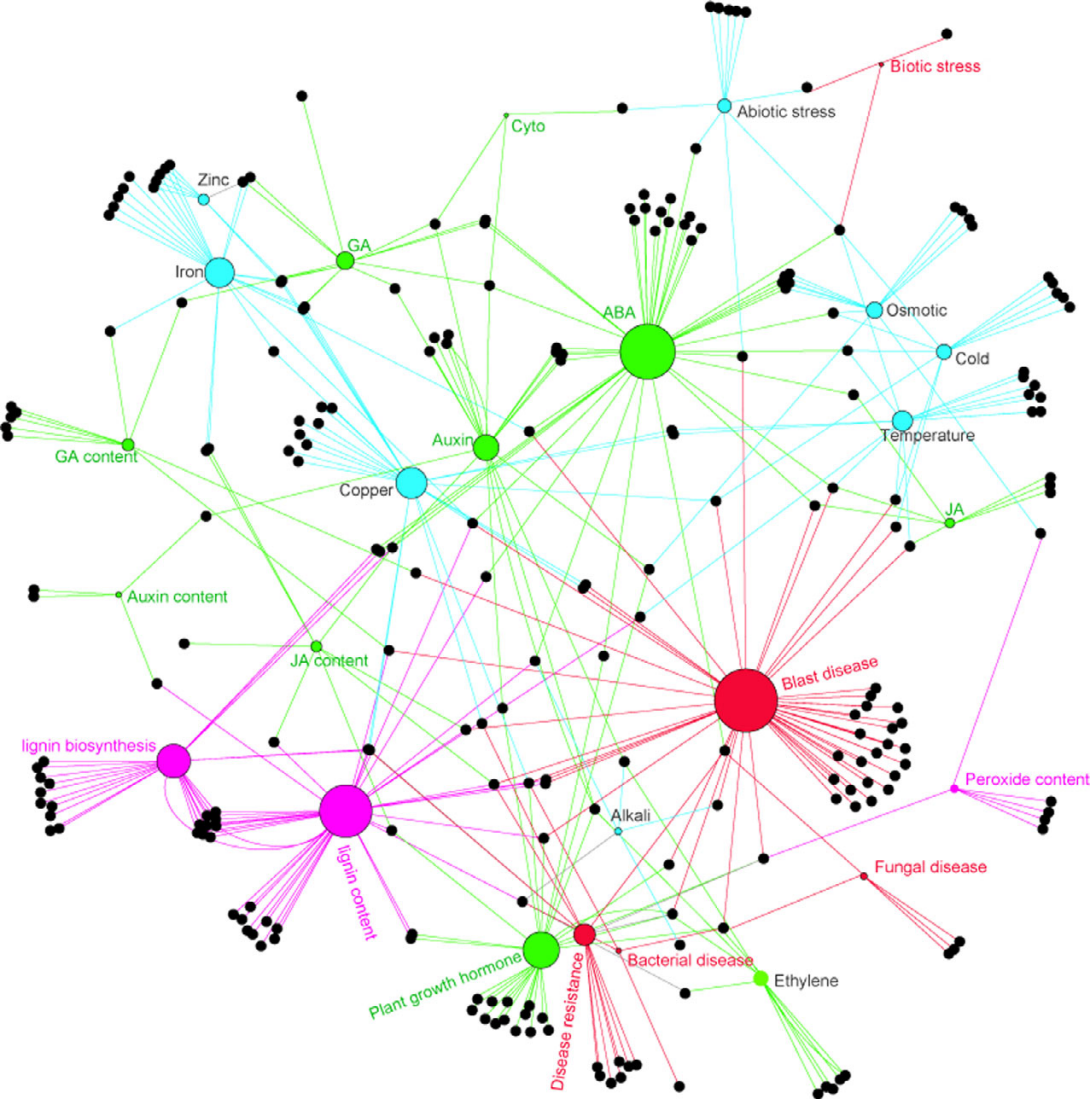
Network of the DEG TO to *H. mucronata.* The black dots, genes, are significantly regulated by *H. mucronata*. Circles filled in purple, green, red and cyan are traits relative to secondary metabolites, hormone, biotic stress and abiotic stress, respectively, and their sizes are relative to the total number of genes.

To explain the DEG functions and interaction between rice and *H. mucronata*, we conducted TO enrichment analysis using clusterprofiler with default parameters (Table S7) 19. According to the TO annotation terms, such as blast disease, disease resistance, lignin content, and sensitivity to gibberellic acid, abscisic acid, JA, iron, copper and zinc sensitivity were enriched. That revealed that there was a complex crosstalk among the plant hormone, biotic stress and abiotic stress in the host response to the nematode infection (Fig. 6).

## Conclusions

The responses of the resistant (Jiabali) and susceptible (Bawangbian) varieties to *H. mucronata* in the roots at 7 DAI were investigated at the transcriptomic level. There were ~2243. DEGs among the four comparison groups. The expression of genes relative to secondary metabolites, such as phenylpropanoid, lignin, cellulose and hemicellulose, and plant hormones, is significantly higher in the Jiabali than in the Bawangbian variety, which could be the primary reason the Jiabali variety displays significantly greater levels of resistance to *H. mucronata* than Bawangbian, and the results of some genes through quantitative real‐time PCR were similar with that of the transcriptome. The genes related to nutrient elements play important roles in improving the resistance of rice to *H. mucronata*.

## Conflict of interest

The authors declare no conflict of interest.

## Author contributions

XtS and RC conceived and designed research. LZ mainly contributed to RNA‐seq analysis. LZ, ZT and XS contributed to data collection. XS and JM analyzed all the data. XtS wrote the manuscript. JM and RC contributed to revising the manuscript. All authors read and approved the manuscript.

## Supporting information


**Table S1**. Primers used for quantitative real‐time PCR.
**Table S2**. Overview of the sequencing data obtained and the mapping of these sequences to the rice genome.
**Table S3**. The novel genes in the roots of the resistant (R51) and susceptible (R155) varieties.
**Table S4**. (A) Annotations of the total of DEGs. (B) Change of the DEGs in GSE35843. (C) MapMan annotation of DEGs. (D) Rice Oryzabase annotation of the DEGs.
**Table S5**. Total GO annotation of the DEGs from the roots of the resistant and susceptible variety at 7 DAI with the corrected *P* < 0.05 in the four groups.
**Table S6**. DEGs of the simultaneous change at 7 DAI.
**Table S7**. TO enrichment analysis of the DEGs.

## Data Availability

The raw sequencing data have been deposited in the National Center for Biotechnology Information database under BioProject accession code PRJNA491309.
